# Is computed tomography of the head justified in patients with minor head trauma presenting with Glasgow Coma Scale 15/15?

**DOI:** 10.4102/sajr.v22i1.1329

**Published:** 2018-09-27

**Authors:** Chuma Singata, Sally Candy

**Affiliations:** 1 Department of Radiology, Faculty of Health Sciences, University of Cape Town, South Africa; 2 Department of Radiology, Groote Schuur Hospital, University of Cape Town, South Africa

## Abstract

**Background:**

In keeping with radiology departments in tertiary referral hospitals in developing countries offering computed tomography (CT) head scan services, the radiology department at Groote Schuur Hospital (GSH) in the Western Cape of South Africa undertakes several such scans annually. Of these scans, many are undertaken for post-trauma patients with minor head injury (MHI). While there is agreement that MHI patients with Glasgow Coma Scale (GCS) scores of 13–14/15 may well benefit, there is doubt as to the clinical utility of routine CT head scanning in MHI patients with GCS scores of 15/15.

**Objectives:**

This retrospective descriptive study of patient records was undertaken to determine the frequency and clinical significance of any abnormalities found on CT head scans of 460 patients with MHI and GCS scores of 15/15, scanned at GSH between 2012 and 2014.

**Method:**

Ethical clearance was obtained and the records of 460 MHI patients with GCS scores of 15/15, loss of consciousness (LOC) and amnesia who underwent CT head scanning at GSH between 2012 and 2014 were then retrieved from the Philips picture archiving and communication system (PACS). Patient records, containing illegible referral forms or technically inadequate CT head scans, were excluded from the study. Patients’ biographical, clinical and CT head scan data were entered into sequentially numbered data collection forms. These data were tabulated and summed as percentage distributions. Patients’ CT head scan findings were reviewed and classified as either showing normal or abnormal features. Abnormalities detected on CT head scans were classified as being either clinically significant or clinically non-significant.

**Results:**

Referral forms and CT scan reports were obtained for 460 MHI patients from a sample of 497 patients, calculated by using the equation for estimating a single proportion from a large sample (precision 1.5%). The sample obtained yielded an acceptable response rate of 460/497 (92.6%). Of 460 (100%) scan reports, 320 (69.6%) showed no abnormality, while 140 (30.4%) showed abnormality. Of the 140 abnormal scans, 107 (23.3%) showed clinically non-significant abnormality, while 33 (7.2%) revealed clinically significant abnormality. Twenty-two (4.8%) of these clinically significant scans showed brain contusion and 11(2.4%) showed skull fracture. No subdural or extradural haematoma, shift or herniation were reported and none of the 33 patients whose CT scans showed clinically significant abnormality underwent urgent neurosurgical intervention.

**Conclusion:**

Of the 460 CT head scans performed at GSH for MHI with LOC but normal GCS between 2012 and 2014, none required urgent neurosurgical intervention. This is in accordance with the 2012 Kimberley Hospital Rule (KHR), a management protocol which indicates that CT head scanning in patients with MHI and GCS scores of 15/15 can safely be delayed for 8 h. An audit of the records of patients excluded from this study coupled with an analysis of data from other Western Cape hospital CT head scan databases could help ensure that this scarce resource is used cost-beneficially for all head-injured patients in the Western Cape catchment area.

## Introduction

Computed tomography (CT) is an expensive but valuable clinical resource which should be judiciously used to ensure that all patients who might benefit from CT head scanning are scanned timeously. The use of CT scanning in resource-constrained developing countries, such as South Africa, should be informed by clear guidelines that ensure that CT is used cost-beneficially. This is especially true where the practice of defensive medicine by referring clinicians drives up the demand for clinically doubtful CT scans.

Computed tomography scanning for post-trauma patients with head injury constitutes much of the demand for CT scanning in the radiology department at Groote Schuur Hospital (GSH). Groote Schuur Hospital is a level-4 referral hospital with an extensive clinical catchment area. While the injuries patients sustain arise from pedestrian and motor vehicle accidents, falling from heights, gunshot and occupation-related injuries, many of which are associated with alcohol intoxication, the actual scale of such injuries in South Africa is inadequately documented and poorly understood.^1^

A preliminary review of traumatic head injury CT scan request forms received in the GSH radiology department showed that many requests were made for patients with minor head injury (MHI). Minor head injury is currently defined as head injury that results in loss of consciousness (LOC) for up to 30 min and post-traumatic amnesia (PTA) in patients presenting with a Glasgow Coma Scale (GCS) score of 13–15.^2,3^

The GCS, famously introduced in 1974 by a Glasgow working group, is used to objectively record a patient’s level of consciousness and neurological functioning at the bedside in a way that can readily be communicated between clinical staff. There is now a body of published evidence demonstrating the clinical value of the GCS, and it is used in most clinical settings.

Several authors have argued that because patients with GCS scores of 13 and 14 have more severe injuries than patients with GCS scores of 15, patients with GCS scores of 15 should be regarded as a separate category and be termed ‘minor head injury’.^4,5,6,7^

At GSH radiology department, it is evident that a high proportion of post-traumatic head injury CT scan requests are made for patients with minor head injuries and GCS scores of 15. This finding raised the question of whether or not the scarce CT resource is being used optimally. This led to the present study being conducted to investigate whether or not the CT head scanning of patients with head injury, a GCS score of 15/15 and no focal neurological deficit would lead to an alteration in the medical or surgical management of the patient.

Much work has been undertaken to develop decision rules to govern the use of CT scanning in patients with MHI in order to assist clinicians to triage patients after head injury. Such work has mainly been carried out in developed world settings where resources are more freely available than they are in the developing world. The computed tomography in head-injured patients (CHIP) protocol is one such set of guidelines.^8^ There is a particular need for guidelines in remote settings where the transportation of head-injured patients to centres where CT can be undertaken is notably difficult.

In 2012, the Kimberley Hospital Rule (KHR) – derived from the National Institute for Health and Clinical Excellence (NICE) – achieved 95% sensitivity and 45% specificity.^9^ The KHR which allows patients with a GCS score of 15 and a history of LOC to be scanned semi-urgently (within 8 h) should be of value in both developed and developing settings. This 8 h rule should assist clinicians to triage patients with head injury, by ensuring that those most in need are scanned soonest, and those patients whose scans are less urgent are scanned later. Such triage should adjust the pace at which CT scans are conducted and allow departments to better manage this resource.^10^

## Method

This retrospective descriptive study of patients’ records and CT brain scan findings was undertaken to determine the frequency and clinical significance of any abnormalities found on the CT head scans of 460 patients with MHI referred to the GSH radiology department between 2012 and 2014. A MHI patient was defined as a patient having a history of LOC, PTA and a GCS score of 15/15 following an episode of head trauma.

After approval had been gained from the Human Research Ethics Committee of the University of Cape Town (HREC REF: 098/2015), the CT request forms and patient reports of 460 patients who underwent CT head scanning over the period 2012–2014 were retrieved from the Philips picture archiving and communication system (PACS). The selected patients included in the study all had a GCS score of 15/15, a history of LOC and/or PTA. Patients were included in the study even if they had multisystem trauma, anticoagulant use or evidence of drug or alcohol use, provided they had a GCS score of 15/15.

The sample size was calculated at 497 using the equation for estimating a single proportion from a large sample with an acceptable precision of 1.5%. Patients whose records revealed any of the following characteristics were excluded from the study: Children aged less than 13 years; illegible CT request forms or inadequate reports; inadequate CT scans whether owing to movement, artefact or the incorrect window; reports not reviewed by a senior radiologist; patients with penetrating trauma; clinically palpable or radiographically detected skull fracture; and clinical seizure or focal neurological abnormality.

Patients were allocated consecutive numbers, and information from their records was then entered into a data recording form. Categorical data, including age, sex, LOC, PTA, indications of trauma, namely, ecchymosis, abrasion, or swelling, were recorded. In addition patients’ symptoms including nausea, vomiting, headache and dizziness were also recorded.

## Computed tomography scan classification

Computed tomography scans were considered abnormal if they exhibited any of the following characteristics: subdural, epidural or subarachnoid haematoma; parenchymal contusions; cerebral oedema; cerebral herniation (midline shift, uncal or transtentorial); pneumocranium; or skull fracture.

Patients whose CT scans showed abnormal findings were classed as having non-significant abnormality if their scan revealed ecchymosis, scalp swelling and linear skull fracture. Significant CT findings included subdural and extradural haematoma, contusion, cerebral oedema, cerebral herniation, hydrocephalus and depressed or basal skull fracture not detected clinically or on prior skull radiograph.

Head CT scans were conducted either on a 6-slice Siemens or on a 120-slice Toshiba helical scanner. Bone, brain and epidural window views were obtained for all patients.

The data from the data collection sheets were collated, tabulated, summed and presented as percentage distributions as shown in the following section.

## Results

The records of 460 patients were extracted from the GSH radiology department’s PACS against the sample of 497 records calculated using the equation for estimating a single proportion from a large sample with a precision of 1.5%. This yielded an acceptable response rate of 460/497(92.6%).

**FIGURE 1:**
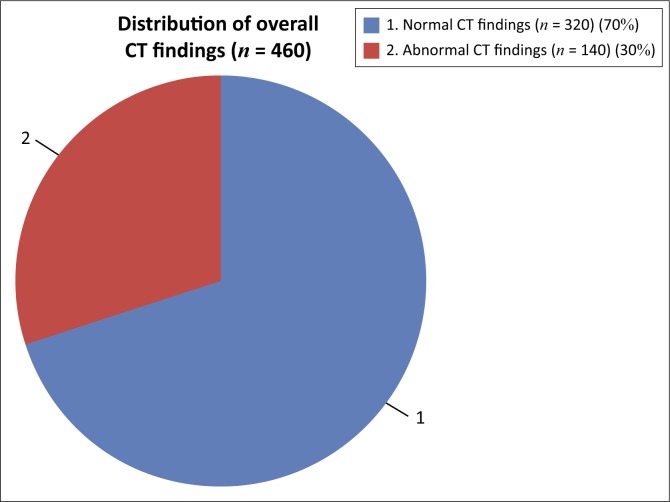
Pie chart demonstrating the distribution of normal and abnormal findings on computed tomography in our sample of patients (*n* = 460).

An analysis of the CT 460 head scan records is given in Figure 1.

**FIGURE 2:**
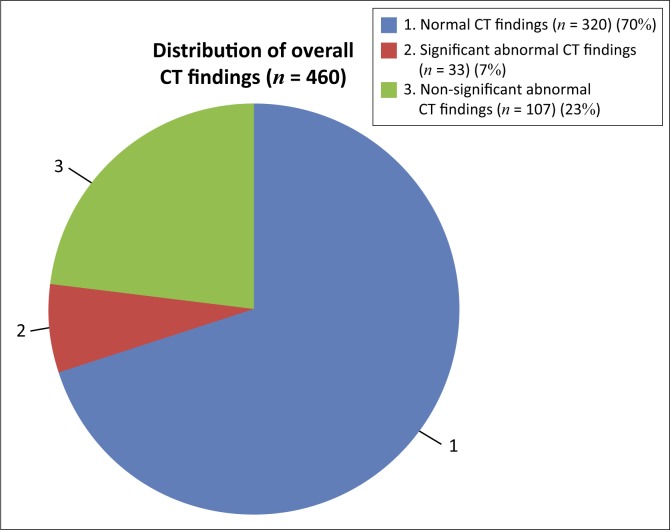
Pie chart differentiation of the abnormal computed tomography brain scans with significant and insignificant findings (*n* = 460).

An analysis of the 140 (30%) CT head scans showing abnormality is given in Figure 2.

Of the total, 33 clinically significant abnormal CT scans, 22 scans revealed brain contusion, while 11 revealed depressed skull fractures and seven showed base of skull fractures. None of the 33 abnormal CT scans revealed the presence of serious abnormalities such as cerebral herniation, extradural collection, subdural collection or cerebral oedema – abnormalities that would have necessitated urgent neurosurgical intervention. (Table 1)

**TABLE 1:** Analysis of findings in 33 (7.2%) computed tomography scans showing clinically significant abnormalities.

Analysis of findings	*n*	%
Number of scans showing brain contusion	22	4.8
Number of scans showing skull fracture	11	2.4
Number of scans showing an indication for urgent neurosurgical intervention	0	0
Total number of scans showing significant abnormality	33	7.2

The records of the 33 patients whose CT scans showed significant abnormality were reviewed to determine whether these patients had reported symptoms known to indicate serious head injury: headache, dizziness, vomiting or nausea. These findings are documented in Table 2.

**TABLE 2:** Number of patients reporting clinically alerting symptoms.

Patients	*n*	%
Number of patients reporting clinically alerting symptoms	19	4.1
Number of patients denying clinically alerting symptoms	14	3.0
Total number of patients with clinically significant CT abnormality	33	7.2

CT, computed tomography

Of the 19 patients who experienced symptoms, seven had reported dizziness, five had vomited and seven had headache.

## Discussion

This study revealed that, of 460 patients who had traumatic head injury with LOC and/or amnesia but with a GCS score of 15 at presentation, 33 (7%) had clinically significant CT head scan findings. Contusion and depressed skull fracture were the most common significant positive findings in 22 (4.8%) and 11 (2.4%) patients, respectively; 7(1.5%) patients had base of skull fractures. No patient had subdural or epidural haematoma or cerebral herniation requiring urgent neurosurgical intervention.

These findings accord with several previous studies. A large prospective study (1382 patients) conducted by Miller et al. concluded that procedural CT of the brain in patients with a GCS score of 15 after MHI had minimal significance and was not justifiable.^11^ Stein & Ross et al., in a study designed to examine the same question, concluded that only patients with a GCS score of less than 15 warrant immediate and/or urgent CT brain scanning.^12^ Davis et al. after reviewing the CT brains in children with MHI and LOC found a number of CT abnormalities similar to those found in this study, though they reported more intracranial haemorrhages (8% vs. 4.7%). Their report did not specify the type of haemorrhage found or whether these patients required neurosurgical intervention, but they concluded that CT is a safe and cost-effective means of reducing patient admission for overnight observation and that it significantly reduces the cost incurred by missed or delayed head injury management.^13^

However, in a prospective study of 712 trauma patients with GCS score of 15, Jeret et al. (1993) found that 67 patients had intracranial traumatic lesions, two of whom required neurosurgical intervention and one who died. These authors concluded that there is no risk factor or clinical predictor occurring in isolation or in combination that can provide an accurate prediction of CT abnormality/normality, or that can alter the algorithm of imaging and the management of MHI.^5^

Regarding patients’ symptoms, Hsiang et al. (1997) reported a high correlation between headache and MHI.^5^ Nausea and vomiting are however non-specific symptoms that may result from alcohol intoxication and substance abuse, as well as from head injury. In our study, patients with significant findings on CT also reported a higher incidence of vomiting (15%) and headache (21%), than those with insignificant or negative CT findings. This retrospective study of stored patient records did not allow the investigator to determine the clinical history and neurological outcome of patients whose CT scans revealed significant abnormality.

Computed tomography head decision rules have been mainly formulated in developed countries where the trauma burden, staffing and financial pressures are different from those prevailing in developing countries such as South Africa. With this proviso in mind, the KHR proposed by Bezuidenhout and colleagues in 2013 warrants careful consideration.^9^ Modified from the well-known National Institute for Health and Care Excellence (NICE) guideline, this offers a single unifying rule that is ideal for a resource-limited environment with high prevalence of trauma. Emergency scans are resource-intensive. With 90% sensitivity and 45% specificity in the detection of clinically significant intracranial findings, the KHR aims to rationalise the timing of emergency CT brains without reducing the number of scans performed. Based on this rule, patients with a normal mental status (GCS 15) but with a history of LOC and/or amnesia should undergo CT scanning within 8 h. In the Western Cape, this would allow the patient to be observed at a primary treatment centre and to be transferred for a CT head scan at a tertiary level referral centre within 8 h.

The absence of any patient requiring neurosurgical evacuation of an intracranial haematoma in this study justifies cautious observation in this patient group. The findings in the current study indicate that patients with minor head trauma could be safely managed at primary- or secondary-level units. In busy tertiary trauma units, the reduction in patient numbers could improve the management of those patients with unequivocal head injury.

An unpublished study conducted at GSH indicated that the time from a patient being injured to their undergoing an initial CT scan is an astounding 18 h.^13^ This staggering delay is attributable mainly to the unavoidable constraints which result from the Western Cape having an overtaxed public-sector ambulance service. A service which cannot cope with the demands placed on it for the safe transport of trauma victims from primary- and secondary-level facilities solely for CT head scans.

Increasingly, CT scanners are being installed at secondary-level hospitals in South Africa but are not staffed outside of normal working hours (08:00 to 16:00). This study suggests that patients who fit the criteria for MHI with a presentation GCS score of 15/15 could safely be managed at the referral hospital awaiting next-day scan. This would have a significant positive impact on heavily burdened ambulance services and on the trauma units at the major tertiary centres.

An analysis of the actual cost of the CT head scans conducted at GSH radiology department should be undertaken to allow us to fully understand the work of the department. When a contusion is detected on a patient’s CT head scan, that patient is generally admitted to hospital for neurological observations for a variable period of time. A follow-up CT is often performed to exclude radiological change, even in the absence of neurological deterioration. Such patient admissions invariably increase the financial and staffing burden in overstretched neurological units.

## Limitations of the study

- The data obtained in this retrospective study were limited to the collection of data from patient CT scan request forms submitted by clinicians and stored in GSH, RIS and PACS.
- No files or clinical notes were reviewed; hence, the type of neurosurgical management and the eventual clinical outcome of these MHI patients were not determined.
- Variables such as length of LOC may have been inaccurately estimated and the mode of injury was not always documented.
- The sample size was smaller than many similar studies. We had a shortfall of 35 patients on our recommended calculated sample size. Small sample size and different patient demographic and socio-economic factors may play a major role in the different outcomes.
- This study was confined to the CT scan reports of patients presenting to a single urban tertiary hospital.
- No further review of the records of patients with illegible records or doubtful CT scan reports was undertaken, and the scale of this aspect of the record review was not estimated.

## Future applications

Currently, patients presenting post-trauma with normal GCS scores to the radiology department at GSH are imaged urgently in the face of CT resource scarcity. In the light of the findings of this retrospective study, we recommend a large prospective study of patients presenting with head trauma but with a GCS score of 15. This would be designed to include detailed documentation of the mechanism of injury and other associated injuries (including, but not restricted to, suspected cervical spine injuries). A prospective study could be invaluable in helping the radiology service to formulate a safe policy for the management of MHI patients. However, a further review of the records of patients who have undergone CT scans for MHI in other regional referral centres, such as Tygerberg Hospital (TBH), for example, could yield further insight into this problem at little extra cost. In addition, an audit of the use of CT scan at GSH radiology department for MHI could possibly help the department to use this scarce resource more efficiently as would an audit of the records of patients who underwent CT head scan but whose records were excluded from this study.

Computed tomography head decision rules have proved effective in other countries with reliable sensitivity and specificity. To curb overutilisation of this scarce resource, a trial of the KHR at GSH radiology department is warranted.

## Conclusion and recommendations

This study showed that most CT scans performed in patients who had experienced LOC after head trauma but who had recovered to normal mental status (GCS score of 15 and no focal neurology) were normal. No scans with herniation and sub- or epidural haematoma were identified. However, a small number of patients had significant findings, such as intracranial haemorrhage and depressed skull fracture even in the face of a normal level of consciousness and normal neurology. A normal level of consciousness (GCS score of 15) coupled with absence of neurological deficit does not therefore exclude the possibility of the patient having significant traumatic brain injury or eliminate the need for a CT scan imaging in this subgroup of patients.

Although only 33/460 (7.2%) of patients in this study had a significant CT scan finding warranting referral to a tertiary centre, the clinical needs of this group of patients should be addressed. South Africa is a resource-limited country and many health care centres lack trained personnel and may not have CT scanners. This lack of accessibility may delay imaging and essential surgical management. We therefore agree with the KHR recommendation that patients with MHI and a GCS score of 15 should undergo CT scanning but that such scanning can safely be delayed by 8 h. A well conducted prospective study with clearly defined clinical parameters may shed light on the subject. It is likely, however, that medical litigation and the potentially catastrophic outcome of a missed intracranial injury will continue to drive the demand for CT in the setting of MHI.

## References

[1] Nell V, Brown D Epidemiology of traumatic brain injury in Johannesburg—II. Morbidity, mortality and etiology. SocSci Med. 1991;33(3):289–296. 10.1016/0277-9536(91)90363-H 1925693

[2] Shoar S, Saadat S CT Scanning in minor head injury. CT scanning–Techniques and applications InTechOpen; 2011,pp. 161–176, Cape Town.

[3] Heltemes K, Holbrook T, MacGregor A, Galarneau M Blast-related mild traumatic brain injury is associated with a decline in self-rated health amongst US military personnel. Injury. 2012;43(12):1990–1995. 10.1016/j.injury.2011.07.021 21855064

[4] Teasdale G, Jennett B Assessment of coma and impaired consciousness: A practical scale. Lancet. 1974;2:81–84. 10.1016/S0140-6736(74)91639-0 4136544

[5] Hsiang J, Yeung T, Yu A, Poon W High-risk mild head injury. J Neurosurg. 1997;87(2):234–238. 10.3171/jns.1997.87.2.0234 9254086

[6] Teasdale G. Head injury. J NeurolNeurosurgPsychiatr. 1995;58(5):526–539. 10.1136/jnnp.58.5.526 PMC1073480 7745397

[7] Miller E, Derlet R, Kinser D Minor head trauma: Is computed tomography always necessary? Ann Emerg Med. 1996;27(3):290–294. 10.1016/S0196-0644(96)70261-5 8599485

[8] Smits M, Dippel D, Steyerberg E, et al Predicting intracranial traumatic findings on computed tomography in patients with minor head injury: The CHIP prediction rule. Ann Intern Med. 2007;146(6):397–405. 10.7326/0003-4819-146-6-200703200-00004 17371884

[9] Bezuidenhout A, Hurter D, Maydell A, et al. The Kimberley Hospital Rule (KHR) for urgent computed tomography of the brain in a resource-limited environment. S Afr Med J. 2013;103(9):646–651. 10.7196/samj.6876 24300685

[10] Miller E, Holmes J, Derlet R Utilizing clinical factors to reduce head CT scan ordering for minor head trauma patients. J Emerg Med. 1997;15(4):453–457. 10.1016/S0736-4679(97)00071-1 9279694

[11] Stein S, Ross S The value of computed tomographic scans in patients with low-risk head injuries. Neurosurgery. 1990;26(4):638–640. 10.1227/00006123-199004000-00012 2330085

[12] Jeret J, Mandell M, Anziska B, et al. Clinical predictors of abnormality disclosed by Computed Tomography after mild head trauma. Neurosurgery.1993;32(1):9–15. 10.1227/00006123-199301000-00002 8421561

[13] Owen J, Andronikou S Value of follow-up CT in injury assessment (dissertation).University of Cape Town; 2015, Cape Town.

